# Study on the independent effect of thyroid hormone based on uric acid level on NAFLD

**DOI:** 10.1186/s41043-021-00247-w

**Published:** 2021-05-03

**Authors:** Guanqun Chao, Liying Chen

**Affiliations:** Department of General Practice, Sir Run Run Shaw Hospital, Zhejiang University, Hangzhou, 310016 China

**Keywords:** Nonalcoholic fatty liver disease, Uric acid, TSH, FT3, FT4

## Abstract

**Objective:**

This study aims to explain the correlation among non-alcoholic fatty liver disease (NAFLD), hyperuricemia, and thyroid function and to find independent risk factors for each other.

**Methods:**

Data were obtained from subjects who underwent health examination in the Health Promotion Centre of Sir Run Run Shaw Hospital of Zhejiang University from January 2017 to February 2019. The diagnosis of NAFLD was according to the clinical diagnosis of the guidelines. Serum uric acid (SUA) > 360 μmol/L (female) and SUA > 420 μmol/L (male) were enrolled in the hyperuricemia group. R software was used for statistical analysis.

**Results:**

55,449 subjects were included in the analysis. 34.27% of patients were classified as NAFLD group (*N*=19004), and 65.73% of patients were classified as non-NAFLD group (*N*=36445). The levels of gender ratio, age, BMI, waist circumference, systolic blood pressure (SBP), diastolic blood pressure (DBP), fasting blood glucose (FBG), HbA1c, triglyceride (TG), high-density lipoprotein (HDLC), alanine aminotransferase (ALT), aspartate aminotransferase (AST), urea nitrogen (BUN), creatinine (CR), FT3, FT4, and TSH were significantly different between the non-NAFLD group and NAFLD group. Age, BMI, waist circumference, DBP, fFBG, HbA1c, total cholesterol (TC), low-density lipoprotein (LDLC), AST, and UA were all independent risk factors for NAFLD. In the normal uric acid group, variables other than SBP and TSH were independent factors of NAFLD. In the hyperuricemia group, all variables except SBP, FT4, and TSH were independent factors of NAFLD.

**Conclusion:**

The level of uric acid is related to the occurrence of NAFLD. Hyperuricemia is one of the independent risk factors of NAFLD. TSH level is not related to the occurrence of NAFLD, while FT3 and FT4 may be related to NAFLD.

## Introduction

Nonalcoholic fatty liver disease (NAFLD) is a common chronic disease characterized by the accumulation of fat in the liver, which can be associated with obesity, and can progress to fibrosis, cirrhosis, and even liver cancer [1]. NAFLD is considered as an important feature of metabolic syndrome and an important factor causing cardiovascular diseases. More studies have suggested that NAFLD is related to male sexual dysfunction and reproductive dysfunction [2]. NAFLD has also been shown to be associated with poor metabolism and oxidative stress [3]. Other studies have linked NAFLD to sedentary behavior and poor eating habits [4]. NAFLD is one of the most common chronic liver diseases in developed countries [5]. Except for lifestyle intervention, no drugs are currently approved for the treatment of NAFLD, so more clinical studies are needed to find new methods for the treatment and management of NAFLD.

Uric acid is mostly metabolized by the kidneys and is a product of purine metabolism in the human body. Hyperuricemia is a common manifestation in patients with chronic kidney disease, which is believed to be related to vascular smooth muscle proliferation, endothelial dysfunction, and interstitial inflammatory infiltration [6]. Studies have shown that hyperuricemia is associated with hypertension, hyperlipidemia, diabetes, and obesity [7]. Hyperuricemia and NAFLD have similar clinical and pathological characteristics, so we speculate that there is a certain relationship between them.

Thyroid hormones T3 and T4 are synthesized in the thyroid gland and are associated with thyroglobulin [8]. Some researchers proposed that TSH, FT3, and BMI presented a nonlinear positive quadratic relationship, while FT4 and BMI presented a negative nonlinear relationship [9]. It has also been found that obese children can significantly improve the structure and function of the thyroid gland after weight loss [10]. There is also a study suggesting that thyroid function is related to cardiovascular events. Compared with those with normal thyroid function, the incidence and mortality of cardiovascular events in those with abnormal thyroid function are significantly increased [11]. Thus, thyroid function is associated with obesity and cardiovascular events. We hypothesized that thyroid hormones might also be associated with NAFLD and hyperuricemia, given their commonalities with metabolic and cardiovascular events associated with NAFLD and hyperuricemia. This study aims to further explain the correlation among NAFLD, hyperuricemia, and thyroid function by collecting data of physical examination population and using statistical analysis method and to find independent risk factors for each other.

## Methods

### Data and methods

Data were obtained from subjects who underwent health examination in the Health Promotion Centre of Sir Run Run Shaw Hospital of Zhejiang University from January 2017 to February 2019. The diagnosis of NAFLD is based on the following three criteria: nonalcoholic, fatty degeneration detected by imaging or histological examination, and other liver diseases excluded [12]. Exclusion subjects: (1) patients with severe cardiac, hepatic and renal insufficiency; (2) recent use of drugs affecting thyroid secretion or previous clear diagnosis of thyroid diseases or use of drugs affecting purine metabolism; (3) liver damage, alcoholism, viral liver disease, alcoholic liver disease, autoimmune liver disease, genetic, and drug-induced liver disease caused by other factors; and (4) malignant tumors and rheumatic diseases. Sir Run Run Shaw Hospital ethics committee reviewed and approved the study.

### Grouping

NAFLD was consistent with no history of alcohol consumption or alcohol consumption equivalent to ethanol < 140 g/week (male), ethanol < 70 g/week (female), except for viral hepatitis, drug-induced liver disease, total parenteral nutrition, hepatolenticular degeneration, autoimmune liver disease, and other specific diseases that can lead to fatty liver disease. According to the diagnostic criteria and exclusion criteria of NAFLD, we divided the samples into NAFLD group and non-NAFLD group.

The results were grouped according to serum uric acid levels. Serum uric acid (SUA) > 360 mmol/L (female) and SUA> 420 mmol/L (male) were enrolled in the hyperuricemia group, while the rest were enrolled in the non-hyperuricemia group.

### Statistical processing

R software was used for statistical analysis. The measurement data conforming to the normal distribution were expressed as the mean ± standard deviation (‾X ± SD). The comparison between the two groups was conducted by *t* test, and *P* < 0.05 was considered statistically significant. Classification data were compared by chi-square test, and the difference was statistically significant by *P* < 0.001. The measurement data of non-normal distribution were expressed by the median (interquartile distance), the Mann-Whitney *U* rank sum test was used for pantwise comparison, and the multivariate logistic regression analysis was used for risk factor analysis.

## Results

### General data and correlation analysis

Finally, 55,449 subjects were included in the analysis. 34.27% of patients were classified as NAFLD group (*N*=19004), and 65.73% of patients were classified as non-NAFLD group (*N*=36445). Gender ratio, age, BMI, waist circumference, systolic blood pressure, diastolic blood pressure, fasting blood glucose, HbA1c, triglyceride, high-density lipoprotein, glutamate transaminase, glutamate transaminase, urea nitrogen, creatinine, FT3, FT4, and TSH were significantly different between the two groups (*P* < 0.05) (see Table 1).

**Table 1:** Baseline characteristics of subjects stratified according to the Non-NAFLD or NAFLD (mean ± SD)

	Non-NAFLD group (*n*=36445)	NAFLD group (*n*=19004)	Overall (*n*=55449)	^a^*P* value
Male gender, %	16,731 (45.9%)	14,587 (76.8%)	31,318 (56.5%)	< 0.001
Age, years	46.0 ±10.7	48.8 ±9.79	47.0 ±10.5	< 0.001
BMI, kg/m^2^	22.9 ±2.84	26.1 ±3.06	24.0 ±3.28	< 0.001
Blood pressure, mmHg				
Systolic blood pressure	119 ±16.2	128 ±15.2	122 ±16.5	< 0.001
Diastolic blood pressure	70.8 ±10.9	77.8 ±10.9	73.2 ±11.3	< 0.001
Waist circumference, cm	79.6 ±8.95	91.6 ±8.23	83.7 ±10.4	< 0.001
FBG, mmol/L	5.12 ±0.885	5.75 ±1.51	5.34 ±1.18	< 0.001
Hba1c, %	5.29 ±0.581	5.67 ±0.923	5.42 ±0.739	< 0.001
Triglycerides, mg/dL	1.37 ±1.03	2.44 ±1.93	1.74 ±1.50	< 0.001
Total cholesterol, mg/dL	4.72 ±0.909	5.02 ±0.996	4.82 ±0.951	< 0.001
High density lipoprotein, mg/dL	1.28 ±0.314	1.07 ±0.242	1.21 ±0.309	< 0.001
Low density lipoprotein, mg/dL	2.67 ±0.733	2.84 ±0.807	2.73 ±0.763	< 0.001
Alanine aminotransferase, IU/L	20.3 ±20.1	35.2 ±27.2	25.4 ±23.9	< 0.001
Aspartate aminotransferase, IU/L	20.1 ±11.1	24.7 ±13.6	21.7 ±12.2	< 0.001
Uric acid, μmol/L	324 ±82.4	397 ±86.1	349 ±90.6	< 0.001
TSH, mIU/L	1.88 ±1.67	1.85 ±1.55	1.87 ±1.63	0.034
FT3, pg/ml	2.59 ±0.895	2.68 ±0.839	2.62 ±0.877	< 0.001
FT4, ng/dl	2.18 ±2.00	2.10 ±1.92	2.15 ±1.97	< 0.001

Note: *BP* blood pressure, *SBP* systolic blood pressure, *DBP* diastolic blood pressure, *WC* waist circumference, *FBG* fast blood glucose, *SUA* serum uric acid, *TC* total cholesterol, *TG* triglycerides, *LDL* low-density lipoprotein, *HDL* high-density lipoprotein, *ALT* alanine aminotransferase, *AST* aspartate aminotransferase, *BUN* blood urea nitrogen, *NAFLD* nonalcoholic fatty liver disease. ^a^Two-sided *P* values for the difference between the non-NAFLD groups and NAFLD groups

### Risk factors for NAFLD group

In order to better determine the correlation between the level of relevant indicators and NAFLD, we conducted logistics regression analysis. In the single-factor regression model, we divided the subjects into two categories, including gender, age, BMI, waist circumference, blood pressure, rapid blood glucose, blood lipid, liver function, uric acid, thyroid hormone, and other relevant indicators for analysis. The results showed that male, age, BMI, waist circumference, diastolic blood pressure, rapid blood glucose, HbA1c, total cholesterol, low-density lipoprotein, AST, uric acid, and FT3 were independent risk factors for NAFLD (OR > 1). In the multivariate logistic regression model, age, BMI, waist circumference, diastolic blood pressure, rapid blood glucose, HbA1c, total cholesterol, low-density lipoprotein, AST, and uric acid were all independent risk factors for NAFLD (OR > 1). Systolic blood pressure and TSH are independent factors (see Table 2).

**Table 2:** Odds ratio of NAFLD incidence by demographic and metabolic factors

Exposure	Univariate		Multivariate	
	OR (95%CI)	*p* value	OR (95%CI)	*p* value
Age	1.026 (1.024–1.028)	< 0.001	1.009 (1.006–1.012)	< 0.001
BMI	1.454 (1.443–1.465)	< 0.001	1.110 (1.098–1.122)	< 0.001
SBP	1.039 (1.038–1.040)	< 0.001	1.000 (0.998–1.002)	0.989
DBP	1.060 (1.059–1.062)	< 0.001	1.016 (1.012–1.019)	< 0.001
WC	1.175 (1.172–1.178)	< 0.001	1.103 (1.098–1.107)	< 0.001
FBG	1.861 (1.818–1.905)	< 0.001	1.120 (1.083–1.158)	< 0.001
HbA1c	2.235 (2.167–2.307)	< 0.001	1.295 (1.230–1.365)	< 0.001
TC	1.392 (1.366–1.419)	< 0.001	0.716 (0.664–0.773)	< 0.001
Hdl-c	0.054 (0.050–0.058)	< 0.001	0.493 (0.435–0.559)	< 0.001
Ldl-c	1.339 (1.308–1.370)	< 0.001	1.738 (1.598–1.892)	< 0.001
AST	1.051 (1.049–1.054)	< 0.001	0.968 (0.964–0.973)	< 0.001
UA	1.010 (1.010–1.010)	< 0.001	1.004 (1.004–1.004)	< 0.001
TSH	0.988 (0.976–0.999)	0.039	0.994 (0.980–1.009)	0.669
FT3	1.122 (1.099–1.146)	< 0.001	0.941 (0.899–0.983)	< 0.001
FT4	0.979 (0.971–0.988)	< 0.001	0.952 (0.934–0.971)	< 0.001

Note: *BP* blood pressure, *SBP* systolic blood pressure, *DBP* diastolic blood pressure, *WC* waist circumference, *FBG* fast blood glucose, *SUA* serum uric acid, *TC* total cholesterol, *LDL* low-density lipoprotein, *HDL* high-density lipoprotein, *AST* aspartate aminotransferase, *BUN* blood urea nitrogen, *NAFLD* nonalcoholic fatty liver disease

### Risk factors for NAFLD group between hyperuricemia group and normal uric acid group

In subjects with the normal uric acid (*N* = 39183), the NAFLD group (*N* = 10615) compared with the non-NAFLD group (*N* = 28568), sex, age, BMI, waist circumference, systolic blood pressure, diastolic blood pressure, triglyceride, total cholesterol, high-density lipoprotein cholesterol (HDL-c), low-density lipoprotein cholesterol (LDL-c), ALT, AST, FT3, FT4, etc., all have significant difference and with no difference of TSH in the two groups (*p* = 0.309). In subjects with the hyperuricemia (*N* = 16266), the fatty liver group (*N* = 8389) compared with the non-NAFLD group (*N* = 7877), sex ratio, age, BMI, waist circumference, systolic pressure, diastolic blood pressure, triglyceride, total cholesterol, HDL-c, LDL-c, ALT, AST, FT3, etc., all have significant difference and with no difference of TSH (*p* = 0.784) and FT4 (*p* = 0.173) in the two groups (see Table 3). Logistic multivariate regression analysis was performed in the two groups respectively. In the normal uric acid group, variables other than systolic blood pressure and TSH were independent factors of NAFLD. In the hyperuricemia group, all variables except systolic blood pressure, FT4, and TSH were independent factors of NAFLD (see Table 4).

**Table 3:** Odds ratio of NAFLD incidence in normal uric acid group and hyperuricemia group

	Normal uric acid			Hyperuricemia		
	Non-NAFLD (*N*=28568)	NAFLD (*N*=10615)	*p* value	Non-NAFLD (*N*=7877)	NAFLD (*N*=8389)	*p* value
Male gender	11,025 (38.6%)	6947 (65.4%)	< 0.001	5706 (72.4%)	7640 (91.1%)	< 0.001
Age	46.0 ±10.6	50.0 ±9.49	< 0.001	46.2 ±11.2	47.4 ±9.98	< 0.001
BMI	22.7 ±2.81	25.8 ±2.97	< 0.001	23.6 ±2.82	26.4 ±3.12	< 0.001
SBP	118 ±16.2	128 ±15.6	< 0.001	122 ±15.8	129 ±14.7	< 0.001
DBP	70.2 ±10.7	76.8 ±10.8	< 0.001	73.2 ±11.0	79.0 ±10.8	< 0.001
WC	78.5 ±8.72	90.2 ±8.30	< 0.001	83.3 ±8.78	93.3 ±7.80	< 0.001
FBG	5.11 ±0.896	5.85 ±1.72	< 0.001	5.17 ±0.841	5.62 ±1.17	< 0.001
HbA1c	5.29 ±0.588	5.75 ±1.02	< 0.001	5.31 ±0.556	5.57 ±0.775	< 0.001
TG	1.28 ±0.898	2.21 ±1.74	< 0.001	1.69 ±1.37	2.72 ±2.12	< 0.001
TC	4.70 ±0.903	4.98 ±0.997	< 0.001	4.79 ±0.929	5.06 ±0.994	< 0.001
Hdl-c	1.31 ±0.313	1.09 ±0.250	< 0.001	1.20 ±0.300	1.03 ±0.227	< 0.001
Ldl-c	2.66 ±0.727	2.85 ±0.804	< 0.001	2.72 ±0.754	2.83 ±0.811	< 0.001
ALT	19.4 ±20.6	32.1 ±26.8	< 0.001	23.3 ±17.9	39.2 ±27.2	< 0.001
AST	19.8 ±11.3	23.6 ±13.5	< 0.001	21.3 ±10.2	26.1 ±13.6	< 0.001
TSH	1.89 ±1.58	1.87 ±1.70	0.309	1.82 ±1.94	1.81 ±1.34	0.748
FT3	2.58 ±0.892	2.68 ±0.830	< 0.001	2.62 ±0.905	2.67 ±0.849	< 0.001
FT4	2.17 ±2.00	2.06 ±1.89	< 0.001	2.18 ±2.00	2.14 ±1.95	0.173

**Table 4:** Odds ratio of NAFLD incidence in normal uric acid group and hyperuricemia group

Exposure	Normal uric acid		Hyperuricemia	
	OR (95% CI)	*p* value	OR (95% CI)	*p* value
Age	1.009 (1.005–1.012)	< 0.001	1.008 (1.004–1.012)	< 0.001
BMI	1.117 (1.102–1.132)	< 0.001	1.108 (1.088–1.128)	< 0.001
SBP	1.002 (1.000–1.005)	0.103	0.996 (0.992–1.000)	0.047
DBP	1.014 (1.010–1.018)	< 0.001	1.021 (1.015–1.026)	< 0.001
WC	1.009 (1.103–1.114)	< 0.001	1.100 (1.092–1.108)	< 0.001
FBG	1.086 (1.044–1.130)	< 0.001	1.173 (1.103–1.248)	< 0.001
HbA1c	1.285 (1.206–1.370)	< 0.001	1.231 (1.125–1.348)	< 0.001
TG	1.602 (1.528–1.681)	< 0.001	1.376 (1.304–1.454)	< 0.001
TC	0.703 (0.639–0.734)	< 0.001	0.734 (0.647–0.833)	< 0.001
Hdl-c	0.483 (0.413–0.564)	< 0.001	0.490 (0.396–0.607)	< 0.001
Ldl-c	1.825 (1.643–2.027)	< 0.001	1.660 (1.443–1.910)	< 0.001
ALT	1.033 (1.030–1.037)	< 0.001	1.040 (1.036–1.044)	< 0.001
AST	0.966 (0.960–0.971)	< 0.001	0.975 (0.968–0.982)	< 0.001
TSH	1.004 (0.986–1.022)	0.659	0.983 (0.960–1.007)	0.158
FT3	0.926 (0.875–0.978)	< 0.001	0.927 (0.856–1.000)	0.056
FT4	0.950 (0.927–0.973)	< 0.001	0.945 (0.912–0.977)	< 0.001

### Analysis of the collinearity of TSH factors

As mentioned above, TSH was not significantly associated with NAFLD in multivariate logistic regression. In order to explore the factors between TSH and NAFLD, we found that after adjusting for age and gender, TSH was associated with NAFLD when other variables were adjusted separately; when TSH was adjusted together with BMI, waist circumference and TG, TSH was not associated with NAFLD (see Table 5).

**Table 5:** Logistic regression for NAFLD outcome with individual risk factors

Adjusted TSH	OR (95% CI)	*P* value
Gender and age	1.029 (1.017–1.041)	< 0.001
BMI	1.011 (0.999–1.025)	0.080
SBP	1.022 (1.010–1.032)	< 0.001
DBP	1.020 (1.008–1.062)	< 0.001
WC	1.009 (0.995–1.023)	0.199
FBG	1.032 (1.019–1.044)	< 0.001
HbA1c	1.028 (1.016–1.041)	< 0.001
TG	1.009 (0.997–1.021)	0.15
TC	1.019 (1.007–1.031)	0.002
Hdl-c	1.034 (1.021–1.046)	< 0.001
Ldl-c	1.025 (1.014–1.037)	< 0.001
ALT	1.016 (1.003–1.029)	0.008
AST	1.019 (1.007–1.031)	0.002
UA	1.017 (1.005–1.029)	0.007
FT3	1.029 (1.017–1.041)	< 0.001
FT4	1.028 (1.017–1.041)	< 0.001

## Discussion

As a common chronic liver disease, NAFLD is on the rise in Asia and is closely associated with liver cancer and mortality [13]. Due to the increasing incidence of obesity and metabolic syndrome, the incidence of NAFLD is also on the rise, leading to cirrhosis and liver cancer [14]. Therefore, NAFLD has become a major global health problem. The investigators [15] noted that NAFLD was associated with components of the metabolic syndrome, including type 2 diabetes, obesity, hypertension, and dyslipidemia. This is consistent with our findings. It is also pointed out that NAFLD is not only related to genetic variation, but also to environmental factors. Our results showed that in addition to blood lipid, blood pressure, and blood sugar, NAFLD was also associated with gender, liver function, thyroid function, uric acid, and other indicators, and the comparison between the two groups had statistical significance. In the past decade, there have been many studies confirming the correlation between uric acid level and metabolic syndrome [16].

It has also been pointed out that uric acid level is an important factor causing metabolic syndrome [7]. In our study, multivariate regression analysis concluded that increased uric acid level was one of the risk factors for NAFLD. This conclusion is consistent with previous studies [17]. Considering that hyperuricemia is a risk factor for fatty liver disease, we consider the following reasons: (1) uric acid is an oxidant that can cause oxidative stress on the liver, thus promoting the development of NAFLD [18]; (2) uric acid may be released by necrotic cells, leading to sterile inflammation, and NAFLD may produce necrotic hepatocytes, which may also be the reason why uric acid is associated with NAFLD [19]; and (3) both hyperuricemia and NAFLD are related to metabolic syndrome, and the reason for the increase of uric acid in metabolic syndrome is that hyperinsulinemia leads to the reduction of uric acid exclusion, so it is speculated that the necessary relationship between uric acid level and NAFLD.

Our study showed that age, BMI, waist circumference, diastolic blood pressure, rapid blood glucose, HbA1c, total cholesterol, low-density lipoprotein, AST, and uric acid were all independent risk factors for NAFLD (OR > 1). Male, total cholesterol, high-density lipoprotein, AST, FT3, and FT4 were protective factors (OR < 1). At the same time, we divided the NAFLD group and the control group according to the uric acid level. In the normal uric acid group, variables other than systolic blood pressure and TSH were independent factors of NAFLD. In the hyperuricemia group, all variables except systolic blood pressure, FT4, and TSH were independent factors of NAFLD.

We found that FT3 and FT4 were considered as protective factors of NAFLD. After secondary grouping, FT3 and FT4 were protective factors for NAFLD under normal uric acid conditions. In the case of increased uric acid, only FT4 was a protective factor for NAFLD. Thyroid hormone has a metabolic effect on protein, carbohydrate, and fat. The decrease of thyroid level is related to the low metabolism, which can be manifested as weight gain, increased blood lipid, and increased blood sugar [20]. Studies have found that thyroxine is related to blood lipids and blood sugar, which can lead to obesity, hyperlipidemia, insulin resistance and other components of metabolic syndrome [21]. One study indicated that the FT3/FT4 ratio was a risk factor for NAFLD, while there was no direct correlation between reduced thyroid function and NAFLD [22]. However, another study confirmed the association between subclinical hypothyroidism and clinical hypothyroidism and NAFLD [23]. Similarly, in a retrospective study, subclinical hypothyroidism was not associated with an increased incidence of NAFLD [24]. In view of the controversial results, the researchers collected the literature and conducted a meta-analysis, concluding that subclinical hypothyroidism was not associated with NAFLD, and thyroid hormone levels were not associated with the presence or absence of NAFLD [25]. We found that TSH was not associated with NAFLD regardless of uric acid levels, consistent with the results of the meta-analysis. However, it was found unexpectedly that FT3 and FT4 were associated with NAFLD, and there were still differences after secondary grouping according to uric acid levels, which were considered as factors. However, the trend of the two levels is opposite, so further prospective studies are needed to further confirm the correlation between thyroid hormone and NAFLD. In addition, thyroid hormone levels are also affected by a variety of factors, including stress, so the results are unstable and controversial.

Since our study found that there was no correlation between TSH and NAFLD, which was inconsistent with other studies, we hoped to look for common clues. We found that after adjusting for age and gender, TSH was associated with NAFLD when other variables were adjusted separately; when TSH was adjusted together with BMI, waist circumference, and TG, TSH was not associated with NAFLD. Therefore, we hypothesized that the correlation between TSH and NAFLD may be affected by body weight and blood lipids, which needs to be further confirmed by further studies. In summary, NAFLD, hyperuricemia, and thyroid dysfunction are all related to components of metabolic syndrome, and hyperuricemia is a risk factor for the increased incidence of NAFLD. Whether uric acid level is normal or not, TSH is not related to NAFLD, while FT3 and FT4 are related to NAFLD, but whether they are the factors of NAFLD remains to be confirmed.

## Conclusion

The level of uric acid is related to the occurrence of NAFLD. Hyperuricemia is one of the independent risk factors of NAFLD. TSH level is not related to the occurrence of NAFLD, while FT3 and FT4 may be related to NAFLD.

## Data Availability

The datasets used and/or analyzed during the current study are available from the corresponding author on reasonable request.

## Abbreviations

NAFLD: Nonalcoholic fatty liver disease
SUA: Serum uric acid
HDL-c: High-density lipoprotein cholesterol
LDL-c: Low-density lipoprotein cholesterol
